# Multimodal Depression Detection Through Conversational Interactions with an Emotion-Aware Social Robot: Pilot Study

**DOI:** 10.2196/84110

**Published:** 2026-04-27

**Authors:** Pu-Yu Liao, Yu-Quan Su, Xiaobei Qian, Yu-Ling Chang, Yun-Hsiang Lee, Li-Chen Fu

**Affiliations:** 1 Graduate Institute of Networking and Multimedia, National Taiwan University, Taipei, Taiwan; 2 Department of Computer Science & Information Engineering, National Taiwan University, No.1, Sec. 4, Roosevelt Road, Taipei, 106319, Taiwan, 886 2 3366 3558; 3 Department of Psychology, National Taiwan University, Taipei, Taiwan; 4 Department of Nursing, National Taiwan University, Taipei, Taiwan

**Keywords:** depression detection, socially assistive robot, SAR, multimodal fusion, synthetic data generation, emotion induction

## Abstract

**Background:**

Depression affects more than 300 million people worldwide and is a leading contributor to the global disease burden. Traditional diagnostic methods, such as structured clinical interviews, are reliable but impractical for frequent or large-scale screening. Self-report tools like the Patient Health Questionnaire-8 (PHQ-8) require disclosure and clinician oversight, limiting accessibility. Recent artificial intelligence–based approaches leverage multimodal behavioral cues (linguistic, acoustic, and visual) for automated depression detection but remain constrained by limited adaptability, scarce annotated data, weak emotional expression in real-world settings, and the high computational cost of deployment of socially assistive robots (SARs).

**Objective:**

This study introduces Depression Social Assistant Robot (DEPRESAR)-Fusion, a lightweight multimodal depression detection framework designed for natural interactions with emotion-aware SARs. The objective of this study was to enhance detection accuracy in everyday conversations while addressing the challenges of data scarcity, weak emotional cues, and computational efficiency.

**Methods:**

DEPRESAR-Fusion integrates acoustic, linguistic, and visual features with an emotion-aware response module powered by large language models to adapt conversational strategies dynamically. To stimulate richer emotional expression, participants were exposed to emotionally evocative videos before SAR interactions. To overcome data scarcity, we augmented training with (1) public depression-related social media corpora and (2) synthetic samples generated via large language models. The proposed multimodal fusion architecture was evaluated on benchmark clinical datasets for both binary depression classification and PHQ-8 regression tasks. Performance was compared against prior multimodal baselines using root mean square error, mean absolute error, and standard classification metrics.

**Results:**

Participants who viewed emotional stimuli before interacting with SARs exhibited significantly higher emotional expressiveness, leading to improved model performance. Regression tasks showed lower root mean square error and mean absolute error, while classification tasks achieved significantly higher accuracy than the nonstimulus condition. DEPRESAR-Fusion outperformed prior multimodal baselines across multiple benchmark datasets, achieving state-of-the-art performance in both binary classification and PHQ-8 regression. The system maintained a lightweight architecture suitable for real-time deployment on SARs.

**Conclusions:**

DEPRESAR-Fusion demonstrates that integrating emotion induction, data augmentation, and lightweight multimodal fusion can enable accurate and scalable depression detection in naturalistic SAR interactions. By bridging the gap between structured clinical assessments and everyday conversations, this approach highlights the potential of SAR-based systems as nonintrusive, artificial intelligence–driven tools for proactive mental health support.

## Introduction

### Background

Depression is a pervasive mental health disorder affecting over 300 million people worldwide and consistently ranks among the leading contributors to the global disease burden [1]. Beyond its psychological toll, depression adversely impacts behavior and physical health, increasing the risk of cardiovascular diseases and other chronic conditions [2]. Early detection and timely intervention are crucial to improving outcomes; however, many individuals avoid professional help due to stigma, shame, or misconceptions [3], leading to delayed treatment and a higher suicide risk [4].

Traditional diagnostic methods, such as structured clinical interviews and self-report questionnaires (eg, Patient Health Questionnaire-8, PHQ-8 [5]), are reliable but limited in scalability. They rely on disclosure, clinician availability, and structured settings, making them impractical for frequent or large-scale screening. These limitations have motivated research into artificial intelligence (AI)–based depression detection, which leverages multimodal behavioral cues—including linguistic, acoustic, and visual signals—to provide scalable and more objective assessments.

Although promising, current AI approaches face several challenges. First, most models are trained on structured clinical interviews and generalize poorly to spontaneous conversations, limiting adaptability. Second, high-quality annotated datasets remain scarce, restricting the ability to capture subtle linguistic and emotional cues. Third, emotional signals in natural settings are often weak or suppressed, reducing sensitivity without external stimulation. Finally, many state-of-the-art architectures are computationally heavy and unsuitable for real-time deployment on resource-constrained platforms such as socially assistive robots (SARs).

Existing research on depression detection primarily relies on 3 modalities: text, audio, and visual. Advances in machine learning and deep learning have improved predictive performance; however, challenges related to data scarcity, feature robustness, and model generalizability continue to limit clinical deployment. Although text remains the most informative modality, incorporating acoustic and visual cues provides complementary behavioral signals, which motivates multimodal approaches.

### Text-Based Depression Detection

Textual analysis plays a central role in automated depression detection, as linguistic content directly reflects psychological states. Prior studies have leveraged word embeddings and semantic representations to capture depression-related language patterns [6], while affective and mental health lexicons have been introduced to enhance emotional interpretability [7]. Analyses of clinical interview datasets consistently show that text-based features outperform acoustic and visual modalities in predictive accuracy [8-10]. This advantage arises from both the respondent’s verbal expressions and the structured conversational context shaped by interviewer prompts, enabling fine-grained semantic and emotional analysis.

### Acoustic- and Vision-Based Depression Detection

Speech-based approaches commonly extract low-level acoustic descriptors, such as Mel-frequency cepstral coefficient (MFCCs) and extended Geneva Minimalistic Acoustic Parameter Set (eGeMAPS), to capture prosodic and voice-quality cues associated with depression [8,9]. Deep learning models, including convolutional neural networks, further learn discriminative speech representations [10]. However, acoustic features are sensitive to speaker variability, background noise, and contextual factors, which hinder generalization.

Visual cues, including facial expressions, gaze patterns, and action units (AUs), provide additional nonverbal indicators of depressive behavior. While these features capture subtle expression dynamics, their reliability is limited in practice, as individuals with depression often exhibit subdued or inconsistent facial activity influenced by personal traits and environmental conditions.

### Multimodal Fusion and Clinical Interview Studies

Multimodal fusion frameworks integrate textual, acoustic, and visual signals to enhance robustness and sensitivity. Empirical evidence from clinical interview studies indicates that although speech and visual cues contribute auxiliary information, textual features remain the dominant predictors [8-10]. Interview transcripts encode not only linguistic content but also interaction patterns and contextual flow, making them essential for depression assessment. Nevertheless, most existing multimodal systems rely on structured interview settings, limiting their adaptability to spontaneous, everyday conversations.

To address weak emotional expression in natural settings, recent work has emphasized emotional reactivity as a key factor in depression [11]. Emotion-induction techniques help elicit observable affective responses, enriching behavioral signals and improving model interpretability.

### Emotion Induction in Depression Research

Depression involves an interaction between mood—a persistent affective state—and emotion, which refers to stimulus-driven responses. A meta-analysis of laboratory studies on major depressive disorder demonstrated attenuated emotional reactivity to both positive and negative stimuli, such as affective images and videos [12]. Emotion-induction methods range from film clips and images to autobiographical recall and immersive environments. Although effective in eliciting transient emotional responses, these approaches often lack ecological validity and are rarely integrated into conversational or cross-cultural AI systems.

### Positioning Within the Related Work Landscape

Recent studies have explored multimodal and affective conversational systems. Large language model (LLM)–based models [13] excel at generating coherent and empathetic dialogue but are typically resource-intensive, lack multimodal perception, and do not incorporate explicit emotion-induction mechanisms. Multimodal fusion frameworks such as Zhang et al [14] focus on integrating multiple modalities but do not prioritize lightweight deployment or hierarchical fusion. Hsieh et al [15] emphasize efficiency but lack support for natural conversation, while Li et al [16] introduce compact multimodal deep fusion architectures without emotion induction or support for free-form dialogue beyond structured interviews.

In contrast, our framework integrates natural conversational interaction, multimodal perception, hierarchical deep fusion, lightweight deployment, and explicit emotion induction. Support for natural conversation enables the analysis of unscripted dialogue; multimodal fusion enhances robustness through complementary signals; lightweight design allows deployment on resource-constrained platforms; deep fusion facilitates cross-modal interaction at multiple levels; and emotion induction actively elicits affective responses, improving detection sensitivity. To position our framework within the existing literature, we compare representative systems across 5 key dimensions: natural conversational support, multimodal fusion, lightweight deployment, hierarchical deep fusion, and emotion induction. As prior work typically focuses on only 1 or 2 of these aspects, Table 1 provides a consolidated comparison to clarify the distinctive contributions of our approach.

**Table 1.** Comparison of representative systems in affective and multimodal depression detection.

	Support natural conversation	Multimodal fusion	Lightweight model (<1 GB)	Deep fusion architecture	Emotion induction
LLM^a^-based [13]	✓	—^b^	—	—	—
Zhang et al [14]	✓	✓	—	—	—
Hsieh et al [15]	—	—	✓	—	—
Li et al [16]	—	✓	✓	✓	—
Ours	✓	✓	✓	✓	✓

a LLM: large language model.

b Not available.

To address these gaps, we propose Depression Social Assistant Robot (DEPRESAR)–Fusion, a lightweight multimodal depression detection framework designed for naturalistic interaction with SARs. The framework is built upon 3 primary objectives. First, it enables emotion-aware SAR interactions by supporting spontaneous, everyday conversations as the foundation for depression detection. Second, it incorporates emotion induction and adaptive engagement mechanisms by integrating evocative video stimuli with an LLM-based conversational response module, thereby enhancing emotional expressiveness and contextual sensitivity during dialogue. Third, it uses a lightweight multimodal deep fusion architecture that combines linguistic, acoustic, and visual features within a resource-efficient design suitable for real-time deployment on SAR platforms.

By bridging the gap between structured clinical assessments and natural conversations, DEPRESAR-Fusion advances scalable, nonintrusive, and emotionally aware AI-driven mental health support.

## Methods

### System Overview

**Figure 1.**
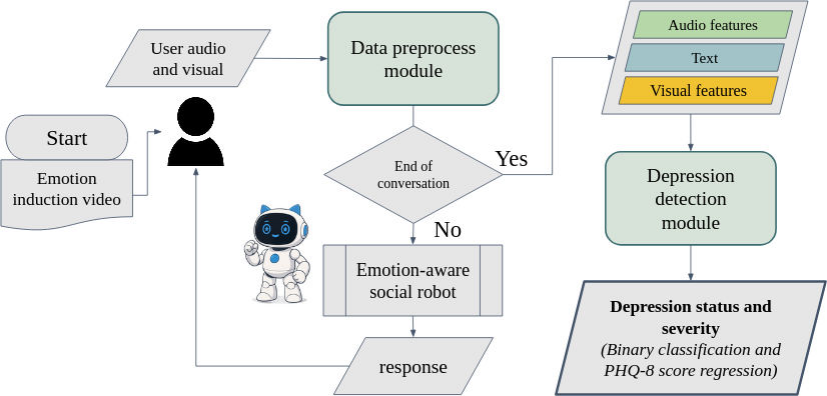
Overview of the Depression Social Assistant Robot–Fusion system, integrating emotion induction, multimodal feature extraction, large language model–guided empathetic dialogue, and depression prediction. PHQ-8: Patient Health Questionnaire-8.

Our proposed system, DEPRESAR-Fusion, was designed for real-time depression detection through natural user interactions (Figure 1). It began with an emotion-induction video to encourage spontaneous emotional responses, followed by a casual conversation with an SAR. During the interaction, multimodal data—including speech, facial expressions, and text—were captured and processed in real time.

Key features were extracted by a data preprocessing module, which encoded audio (eg, MFCCs and eGeMAPS), visual (eg, gaze, head pose, and facial AUs), and transcribed text data. If the user explicitly ended the conversation, the system proceeded to depression prediction. Otherwise, an LLM-powered, emotion-aware social robot dynamically responded via the emotional support conversation (ESC) framework, enhanced with self-consistency prompting to ensure reliable, context-aware, and empathetic interactions. The extracted multimodal features were then passed to a dual-head depression detection module that jointly performed binary classification and PHQ-8 score regression. This architecture enabled simultaneous estimation of depressive presence and severity while maintaining an engaging and supportive user experience.

### Datasets and Depression Assessment

Our experiments were conducted on the Extended Distress Analysis Interview Corpus (E-DAIC), an enhanced version of the Distress Analysis Interview Corpus-Wizard-of-Oz (DAIC-WOZ) dataset, both designed for multimodal depression and anxiety detection from structured clinical interviews. DAIC-WOZ consisted of semistructured interviews conducted via a Wizard-of-Oz setup and included synchronized audio, video, and transcribed speech annotated with psychological assessment scores.

E-DAIC extended DAIC-WOZ with curated splits and standardized multimodal features for machine learning research. It contained 189 interview sessions from 100 participants, providing audio recordings (for acoustic features such as MFCC and eGeMAPS), video recordings (for facial expression and gaze analysis), time-aligned transcripts, and expert-rated PHQ-8 scores as ground truth labels.

Depression severity was measured using the PHQ-8 [5], a validated 8-item instrument ranging from 0 to 24, with scores ≥10 indicating clinically significant depressive symptoms (approximately 88% sensitivity and specificity).

In this study, E-DAIC served as the primary dataset for model training and evaluation, while the DAIC-WOZ benchmark subset was used to assess generalizability and facilitate comparison with prior work.

### Data Augmentation

To enhance model generalization under limited data conditions, we implemented 2 complementary data augmentation strategies involving both text-only and multimodal synthetic data generation.

First, synthetic text-only interview samples were generated using publicly available social media depression datasets, including Reddit Self-reported Depression Diagnosis and Longitudinal Twitter Dataset of Depression in the COVID Era [17,18]. In this pipeline, user posts were segmented into response-style utterances using spaCy to simulate interview dialogue structure. Each segmented sample was temporally aligned to fixed 3-second intervals, while nontextual modalities were padded to maintain input format consistency. Because these datasets provided only binary depression labels, PHQ-8 regression scores were approximated using a thresholding strategy in which depressed samples were assigned a score of 10 and nondepressed samples a score of 0.

Second, synthetic multimodal samples were generated by paraphrasing and restructuring the original structured interview transcripts from E-DAIC [19]. Paraphrasing was performed using ChatGPT (GPT-4o) while preserving the original semantic meaning. Importantly, all paraphrasing was strictly applied only to the training split after dataset partitioning. Test set transcripts were never paraphrased or used in any form during synthetic data generation, ensuring that no semantic leakage occurs. Audio and visual features were resampled from corresponding original samples and temporally aligned with the modified transcripts to construct fully synthetic multimodal instances.

Together, these augmentation strategies improved robustness and enabled the model to better accommodate linguistic variability and multimodal diversity encountered in real-world conversational settings.

### Modality-Specific Processing

The system captured and processed 3 complementary modalities—audio, visual, and text—to extract depression-related features. For the audio modality, acoustic features, including MFCCs and eGeMAPS descriptors, were extracted using openSMILE [20], enabling the capture of prosodic patterns and voice quality characteristics associated with depressive states.

For the visual modality, nonverbal behavioral markers, such as gaze direction, head pose, and facial AUs, were obtained using OpenFace [21]. These features provided cues related to affective expression and social engagement.

For the textual modality, semantic representations were derived from speech transcriptions using pretrained MiniLM [22] and bidirectional encoder representations from transformers [23] embeddings. Both word-level and sentence-level embeddings were incorporated to capture contextual semantics and linguistic indicators of depression.

All extracted features were temporally aligned and encoded to facilitate downstream multimodal fusion and depression assessment.

### Multimodal Fusion

**Figure 2.**
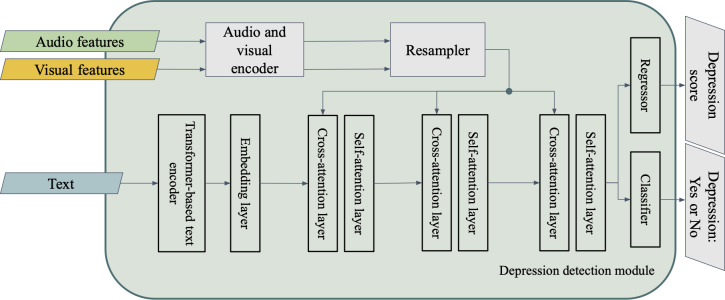
Architecture of the multimodal depression detection module integrating audio, visual, and textual embeddings through attention-based fusion, supporting both binary classification and Patient Health Questionnaire-8 score regression tasks.

To integrate heterogeneous data, the system first normalized and encoded each modality into a shared embedding space. Audio and visual features were transformed using a bag-of-audio-words or bag-of-visual-word framework [24,25], which mapped variable-length sequences into fixed-size distributional vectors via modality-specific codebooks. These normalized embeddings were then temporally aligned and passed to an attention-based fusion module. We used both cross-attention and self-attention mechanisms [26] to model intermodal and intramodal relationships: cross-attention aligned textual features with relevant nonverbal cues, while self-attention captured contextual dependencies within each modality. The fused multimodal representation was then processed by a dual-head architecture (Figure 2). The classification head output the probability of clinical depression, while the regression head predicted a continuous PHQ-8 score. This unified design supported both categorical and continuous interpretations of depression severity, and attention-based fusion enhanced robustness to noise and missing data, improving overall performance. To handle data scarcity in regression targets, we scaled the regression loss.

$L_{reg}$*L*_*reg*_ (around 15), higher than the classification loss *L_cls_* (around 0.5) during training, ensured that the model gave adequate learning attention to the regression head. Training setup—we minimized a multitask objective combining binary cross-entropy (for classification) and mean squared error (for regression): *L*=*α*·*L_cls_*+*β*·*L_reg_* with *α*=1.0, *β*=1.0. We used the Adam optimizer (learning rate 10^–5^, weight decay 10^–4^, batch size 8, and dropout rate 0.1).

### Emotion-Aware Social Robot

#### ESC Framework Overview

The ESC framework divided the emotional support process into 3 stages [27]. This ensured that responses align with the user’s emotional state and the appropriate support strategy. The exploration stage facilitated user expression by prompting open dialogue through questioning, paraphrasing, emotional reflection, and self-disclosure. The comfort stage reinforced empathy and understanding through emotional validation, shared experiences, and supportive affirmations. The action stage focused on resolution strategies through suggestions, practical information, and continued encouragement.

#### Stage Selection via Self-Consistency

Upon receiving user input, the data preprocessing unit transcribed and extracted the last 10 conversational sentences. This context was processed by GPT-4o using a self-consistency method [28] with a temperature of 0.7. This method prompted the model to generate multiple reasoning paths for ESC stage classification (exploration, comfort, and action) and selected the most frequent prediction. Self-consistency improved reliability by reducing variance across LLM outputs.

#### The Full Pipeline

The full pipeline includes the following:

1. User input: Voice, text, and facial expressions were captured by the robot.
2. Preprocessing: The server extracted multimodal features—audio (eg, MFCCs), visual (eg, AUs), and textual content.
3. ESC stage selection: GPT-4o was used to classify the interaction into 1 of the 3 ESC stages using self-consistent prompting.
4. Response generation: Based on the selected stage and strategy, the server generated an empathetic response using GPT-4o at temperature 0°C for consistency.
5. Delivery: The response was returned and delivered by the robot via voice and screen. This integration ensured dynamic and context-aware emotional support, leveraging both multimodal perception and LLM-driven strategies to foster user engagement and enable real-time, supportive interaction.

### Deployment Architecture

DEPRESAR-Fusion operated on a 2-tier architecture: an Android application running on the Kebbi robot by Nuwa Robotics and a remote Windows-based server. This structure balanced real-time interaction with computational efficiency.

#### Kebbi Android Application

The Kebbi robot captured user interactions through voice, facial expressions, and text. It ran the Android front-end, which managed speech recognition, transcriptions, and device expressions. It connected to the Emotion-Aware Social Robot module, with ESC-guided LLM responses generated on the server and spoken aloud by the robot.

#### Windows Server Backend

The core inference pipeline ran on a Windows 10 Enterprise machine (Intel i7-6700, 64GB RAM). It handled preprocessing, multimodal feature extraction, ESC-based reasoning, and depression detection. The server communicated with the robot via an HTTP application programming interface.

### Experimental Setup and Compared Methods

We evaluated the proposed multimodal fusion model for depression detection on the E-DAIC dataset, supplemented with synthetic data derived from the Reddit Self-reported Depression Diagnosis and Longitudinal Twitter Dataset of Depression in the COVID Era datasets to enhance generalizability. Performance was assessed using both classification metrics (eg, *F*_1_-score) and regression metrics (eg, root mean square error [RMSE], mean absolute error [MAE], and concordance correlation coefficient [CCC]) to evaluate binary depression detection and continuous PHQ-8 score prediction, respectively.

To benchmark our approach, we compared it against a diverse set of representative unimodal and multimodal baselines spanning different architectural paradigms. Text-based approaches included the graph convolutional network proposed by Burdisso et al [29], which classified depressive symptoms from interview transcripts, as well as our prior lightweight text-only model (Hsieh et al [15]), which leveraged linguistic signals for depression detection.

Several multimodal deep learning frameworks were also considered. Dham et al [30] integrated audio, visual, and textual features for depression severity estimation, while Li et al [16] proposed a Flexible Parallel Transformer that incorporated handcrafted audiovisual features for enhanced multimodal fusion. Yin et al [31] introduced a hierarchical recurrent neural network designed to capture temporal dependencies across audio, visual, and text modalities, and Zhang et al [14] combined speech and linguistic features for PHQ-8 regression. Additionally, Lau et al [32] developed a parameter-efficient bidirectional long short-term memory with attention, adapted via prefix-tuning for PHQ-8 prediction.

We further evaluated LLMs, including GPT-4o, o1, and o3-mini (OpenAI and Anthropic), under zero-shot settings for textual inference of depressive symptoms.

Together, these baselines cover a broad spectrum of architectures and modality configurations, enabling a rigorous and comprehensive comparison with our proposed lightweight multimodal framework.

### Ethical Considerations

This study protocol was approved by the institutional review board of the National Taiwan University Hospital (202105013RINB), and written informed consent was obtained from all participants prior to participation. To protect participant privacy and confidentiality, all collected data were deidentified and securely stored, and access to the data was restricted to authorized members of the research team only. Participants received NT $200 (US $6.29) as compensation for their participation.

## Results

### Regression Performance

To evaluate the ability of the models to estimate depression severity as a continuous score, we assessed regression performance using 3 standard metrics: RMSE, MAE, and CCC [33]. RMSE captured the average squared difference between predicted and actual PHQ-8 scores, placing greater emphasis on larger errors. MAE measured the average absolute deviation between predictions and ground truth, providing a more interpretable indication of overall prediction error. CCC evaluated both precision and accuracy by assessing the degree to which predicted values conformed to the ground truth in terms of scale and location, thereby offering a more comprehensive measure of agreement.

As shown in Table 2, on the E-DAIC dataset, our proposed model achieved the best RMSE and MAE scores, outperforming all other methods, including those using larger or specialized multimodal architectures. While OpenAI’s o3-mini attained the highest CCC, it did so at the cost of significantly more parameters and computational overhead. In contrast, our model offered competitive concordance with minimal complexity.

**Table 2.** Regression performance on the Extended Distress Analysis Interview Corpus test split. Lower RMSE^a^ or MAE^b^ and higher CCC^c^ indicate better results.

Model (modality)	RMSE	MAE	CCC	Parameters
OpenAI o3-mini (T^d^)	6.1452	4.4909	0.5109	8B (estimated)
Dham et al (A^e^+V^f^) [30]	6.06	5.03	—^g^	5M (estimated)
Li et al (T+A+V) [16]	5.520	4.634	—	—
Yin et al (T+A+V) [31]	5.50	—	0.442	5M (estimated)
Zhang et al (T+AV) [14]	6.11	—	0.403	7M (estimated)
Ours	5.4632	4.3481	0.4501	617K

a RMSE: root mean square error.

b MAE: mean absolute error.

c CCC: concordance correlation coefficient.

d T: text.

e A: audio.

f V: visual.

g Not available.

Table 3 further validates the strength of our method, which achieved state-of-the-art RMSE and MAE results on the DAIC-WOZ benchmark. Notably, our model also achieves the highest CCC (0.6603), suggesting that it better aligns with ground-truth depression scores in both magnitude and scale.

**Table 3.** Regression performance on the Distress Analysis Interview Corpus test split.

Model (modality)	RMSE^a^	MAE^b^	CCC^c^	Parameters
OpenAI o3-mini (T^d^)	5.1281	3.5745	0.6295	8B (estimated)
Zhang et al (T+A^e^+V^f^) [14]	4.66	—^g^	0.560	—
Lau et al (T) [32]	4.67	3.80	—	646K
Hsieh et al (T) [15]	—	3.655	—	745K
Ours (T+A+V)	4.3778	3.3985	0.6603	617K

a RMSE: root mean square error.

b MAE: mean absolute error.

c CCC: concordance correlation coefficient.

d T: text.

e A: audio.

f V: visual.

g Not available.

### Classification Performance

As shown in Table 4, we evaluated the binary classification performance of our proposed method and several baselines on the E-DAIC test split. The objective was to distinguish between depressed and nondepressed individuals based on the standard PHQ-8 cutoff threshold (≥10). Due to the limited public availability of directly comparable classification results on other datasets, we restricted our binary classification evaluation to E-DAIC, which was widely adopted in prior research.

**Table 4.** Classification results on the extended Distress Analysis Interview Corpus test split. Our model outperforms all baselines across all metrics.

Model (modality)	Depression *F*_1_-score	Control *F*_1_-score	Macro *F*_1_-score
OpenAI GPT-4o (T^a^)	0.6316	0.8056	0.7186
OpenAI o1 (T)	0.6667	0.7941	0.7304
OpenAI o3-mini (T)	0.6977	0.8060	0.7518
Burdisso et al (T) [29]	0.63	0.83	0.73
Hsieh et al (T) [15]	0.4722	0.8172	0.6447
Ours (T+A^b^+V^c^)	0.7647	0.8947	0.8297

a T: text.

b A: audio.

c V: visual.

To account for class imbalance, we reported 3 standard *F*_1_-score–based metrics: depression *F*_1_-score, control *F*_1_-score, and macro *F*_1_-score. Depression *F*_1_-score treated the depression class as the positive class, while control *F*_1_-score treated the nondepression (control) class as the positive class. Macro *F*_1_-score was computed as the unweighted average of the depression *F*_1_-score and control *F*_1_-score, providing a balanced evaluation across both classes.

### Analysis

The proposed model achieved state-of-the-art performance in both regression and classification tasks while maintaining an exceptionally small footprint of 617K parameters. On the E-DAIC benchmark, our model attained an RMSE of 5.46 and an MAE of 4.35, outperforming prior multimodal approaches such as Zhang et al [14] (RMSE 6.11) and Yin et al [16] (RMSE 5.50). Similar trends were observed on the DAIC-WOZ dataset, where our method achieved an RMSE of 4.38, an MAE of 3.40, and the highest CCC of 0.66, indicating stronger agreement with ground-truth PHQ-8 scores compared to existing baselines.

In binary classification on the E-DAIC dataset, the proposed framework achieved a macro *F*_1_-score of 0.8297, substantially exceeding both traditional multimodal models and zero-shot LLMs. Notably, the model demonstrated balanced detection capability, with a depression *F*_1_-score of 0.7647 and a control *F*_1_-score of 0.8947, which was critical in clinical screening scenarios to minimize both false negatives and false positives.

Despite its compact size, the proposed model consistently outperformed parameter-heavy LLM baselines such as GPT-4o and o3-mini, which required orders of magnitude more parameters. These quantitative results highlighted three key strengths of our approach: (1) state-of-the-art accuracy with minimal parameters, establishing a new lightweight benchmark for multimodal depression detection; (2) effective joint modeling of regression and classification through a simple multitask 2-head architecture; and (3) robust generalization across both DAIC and E-DAIC datasets, supporting deployment on resource-constrained platforms such as SARs.

### Ablation Study

To quantify the contribution of each modality in our multimodal framework, we performed an ablation study by selectively disabling feature groups during both training and testing. Each ablated modality was replaced with random noise, ensuring that the architecture and learning dynamics remained consistent across settings.

#### Experimental Conditions

We evaluated 5 experimental configurations to analyze the contribution of each modality within the proposed framework. The original configuration activated all modalities, including sentence-level embeddings, word-level embeddings, audio features, and facial features.

In the No SentEmb configuration, sentence-level embeddings were replaced with random noise to remove high-level semantic structure while preserving the remaining modalities. In the No WordEmb setting, word-level embeddings were replaced with noise to eliminate low-level linguistic information.

The No Audio configuration excluded acoustic features, thereby removing prosodic cues such as tone and pitch, which were associated with depressive speech patterns. Similarly, the No Facial configuration omitted visual features, eliminating facial AUs and gaze information that provided nonverbal affective signals.

This ablation design enabled a systematic examination of the relative contributions of semantic, acoustic, and visual modalities to overall depression detection performance.

#### Ablation Study of Modality Configurations

**Figure 3.**
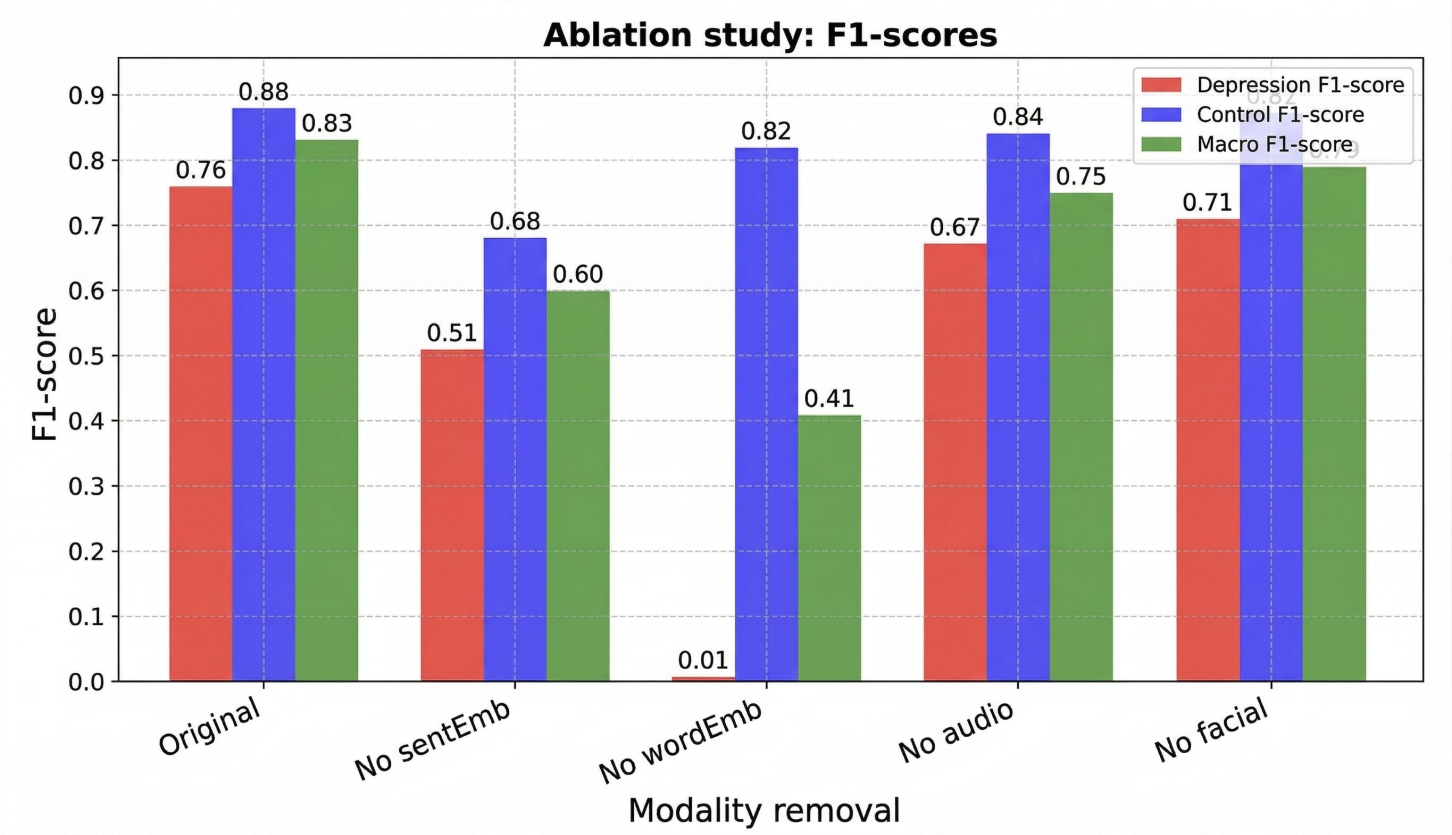
Ablation results across modality configurations. Word-level embeddings show the largest contribution, followed by sentence-level semantics. Audio and visual cues provide additive but less critical information.

As shown in Figure 3, removing word embeddings (No WordEmb) resulted in a substantial degradation in the depression *F*_1_-score, underscoring the essential role of token-level linguistic cues in depression detection. Sentence-level embeddings (No SentEmb) also had a substantial impact, confirming the importance of capturing semantic context. In contrast, removing acoustic (No Audio) or visual (No Facial) features led to moderate declines in overall performance. These findings suggested that while linguistic features were the primary drivers of detection accuracy, audio and visual modalities contributed to supplementary signals that improved robustness. Our results aligned with prior work [34,35], demonstrating the dominant predictive power of text in affective computing tasks. This analysis validated the effectiveness of our multimodal fusion strategy and highlighted the complementary roles of each modality.

### Real-World Evaluation

#### Robot Platform

For the real-world evaluation, the proposed DEPRESAR-Fusion framework was deployed on a commercially available SAR, the Kebbi robot. This robot is equipped with a microphone array for audio capture, an RGB camera for visual input, a display screen for visual feedback, and onboard speech synthesis for spoken responses. Multimodal inputs, including user voice and facial expressions, were captured by the robot and transmitted to a connected server for real-time feature extraction and inference. The Kebbi platform served as the interactive interface through which users engaged naturally with the system.

#### Deployment Setup

To examine the feasibility of real-time deployment, we conducted a pilot user study involving 22 adult participants recruited through the Department of Psychology at National Taiwan University. Participants ranged in age from 19 to 55 years, with the majority between 19 and 25 years old. The gender distribution was balanced (n=11 male participants and n=11 female participants). All participants provided informed consent prior to participation.

Each participant interacted naturally with the Kebbi robot in a spoken dialogue consisting of at least 10 user-generated utterances. Following the interaction, participants completed the PHQ-8, which served as the ground truth measure of depressive symptom severity.

Participants were randomly assigned to 1 of 2 conditions. The emotion-induced group (n=11) viewed a targeted emotion-induction video designed to elicit affective expression prior to the interaction, whereas the control group (n=11) proceeded directly to the robot interaction without priming.

This deployment was designed as a feasibility pilot study to evaluate real-time integration and interaction robustness, rather than to provide statistically powered clinical validation.

#### Statistical Consistency with Benchmark Dataset

To validate the external validity of our deployment, we compared PHQ-8 statistics between the DAIC-WOZ test split and our real-world sample. The class distribution (depressed vs nondepressed) and score statistics (mean and SD) show strong alignment between the 2 datasets (Tables5  6).

**Table 5.** Number of participants with depression and participants without depression.

	DAIC-WOZ^a^ test split	Real-world data
Participants without depression	33	14
Participants with depression	14	8

a DAIC-WOZ: Distress Analysis Interview Corpus-Wizard-of-Oz.

**Table 6.** PHQ-8^a^ score statistics.

Statistics	DAIC-WOZ^b^ test split, mean (SD)	Real-world data, mean (SD)
Average PHQ-8 score	6.98 (6.47)	7.45 (6.40)
Average score (nondepressed)	3.36 (3.04)	3.50 (2.71)
Average score (depressed)	15.50 (3.76)	14.38 (4.44)

a PHQ-8: Patient Health Questionnaire-8.

b DAIC-WOZ: Distress Analysis Interview Corpus-Wizard-of-Oz.

The average PHQ-8 score and SD in both datasets were nearly identical, indicating that our field data were statistically comparable to the DAIC-WOZ benchmark and suitable for generalization assessment.

#### Model Performance in the Wild

We evaluated the model’s classification and regression performance in a real-world setting and compared it to its performance on the DAIC-WOZ test set. The classification model generalizes well to real-world conversations, achieving slightly improved *F*_1_-scores and recall scores, despite a minor drop in overall accuracy to 0.7895 (statistically significant at paired *t* test, *P*=.02). In contrast, regression accuracy drops more noticeably, indicating that real-world data introduce greater variability in symptom expression than observed during training (Tables 7-9).

**Table 7.** Classification task performance.

Classification task	Precision	Recall	*F*_1_-score	Accuracy
DAIC^a^ test split	0.6667	0.8571	0.7500	0.8300
Real-world data	0.7000	0.8750	0.7778	0.7895

a DAIC: Distress Analysis Interview Corpus.

**Table 8.** Regression task performance.

Real-world data	RMSE^a^	MAE^b^
DAIC^c^ test split	4.3778	3.3985
Real-world data	6.5252	4.7217

a RMSE: root mean square error.

b MAE: mean absolute error.

c DAIC: Distress Analysis Interview Corpus.

**Table 9.** Real-world performance with and without emotion induction.

Metric	With emotion induction	Without emotion induction
Regression task		
RMSE^a^	5.0868	7.7650
MAE^b^	3.8653	5.4853
Classification task		
Precision	0.6000	0.6667
Recall	1.0000	0.8000
*F*_1_-score	0.7500	0.7273
Accuracy	0.8182	0.7273
*P* value (Binomial test)	.03	.11

a RMSE: root mean square error.

b MAE: mean absolute error.

#### Impact of Emotion Induction

We further examined the influence of emotion induction on model performance by comparing results across the emotion-induced and control groups. Participants exposed to emotional stimuli before the interaction exhibited improved regression accuracy (significantly lower RMSE and MAE) and stronger classification performance. This improvement reflects the effectiveness of the proposed emotion-induction procedure as a whole, rather than isolating emotion-specific causal effects. Although the observed difference reached statistical significance (*P*=.033), this result should be interpreted with caution given the small sample size. The finding was intended to highlight a preliminary trend rather than to establish a definitive causal conclusion.

#### User Feedback

We conducted a postinteraction survey with 12 Likert-scale questions (6 positive and 6 negative) to assess user experience (Table 10). Figure S1 in Multimedia Appendix 1 summarizes mean ratings across participants, with error bars indicating the SEM. Positive items (Q1-Q6) received generally higher scores than negative ones (Q7-Q12), indicating favorable perceptions of the agent’s understanding, empathy, and clarity—especially Q3 (“I can understand what the robot says”) and Q1 (perceived understanding). Negative items, particularly Q9 and Q10, scored low, reflecting disagreement with negative experiences. The paired positive or negative results confirm effective communication and emotional responsiveness. However, lower scores on Q5 and Q6 (emotional comfort or support) indicated a discrepancy between perceived understanding and perceived emotional comfort. This discrepancy was not unexpected in human-robot interaction settings. While the ESC framework guided the agent to adopt appropriate high-level empathetic strategies, perceived emotional comfort was also strongly influenced by factors such as vocal prosody, response timing, and nonverbal cues. These aspects were only partially addressed in the implementation, which may explain the observed gap between perceived understanding and emotional comfort.

**Table 10.** User feedback questionnaire items.

ID	Question (translated from Chinese)
Q1	I feel the robot understands what I say.
Q2	I feel the robot understands my emotional state.
Q3	I can understand what the robot says.
Q4	I feel the robot shows empathy.
Q5	I feel the robot comforts me.
Q6	I feel the robot fulfills my emotional support needs.
Q7	I feel the robot does not understand what I want to express.
Q8	I feel the robot cannot understand my emotional state.
Q9	I cannot understand what the robot is trying to express.
Q10	I feel the robot lacks empathy.
Q11	I feel the robot does not comfort me.
Q12	I feel the robot cannot fulfill my emotional support needs.

This user feedback provided empirical evidence that our emotion-aware agent not only achieved effective basic communication but also demonstrated empathetic engagement, a critical factor in human-robot interaction. Unlike prior work that often focused solely on task performance, our results emphasized the agent’s ability to foster emotional rapport, which was essential for applications in mental health support. The identified gaps in emotional comfort indicated promising directions for refining conversational tone to enhance user trust and acceptance, advancing the design of socially intelligent agents.

## Discussion

### Principal Findings

This study introduces DEPRESAR-Fusion, a lightweight multimodal depression detection framework designed for naturalistic interaction with SARs. Across both regression and classification tasks, the proposed model demonstrated strong predictive performance while maintaining an exceptionally compact architecture (617K parameters).

On PHQ-8 regression tasks, the model achieved the lowest RMSE and MAE on the E-DAIC dataset and state-of-the-art performance on the DAIC-WOZ benchmark, while maintaining competitive CCC values. These results indicate that the proposed dual-head architecture can effectively estimate both depressive severity and categorical status within a unified framework.

In binary classification, the model achieved a macro *F*_1_-score of 0.8297 on E-DAIC, demonstrating balanced performance across depressed and nondepressed classes. Importantly, the system maintained stable performance during real-world deployment on a commercial SAR platform, suggesting practical feasibility beyond controlled benchmark settings.

The ablation study revealed that linguistic features—particularly word-level embeddings—contribute the most substantial predictive signal. Acoustic and visual modalities provided complementary improvements, enhancing robustness without dominating the predictive process. Furthermore, the pilot deployment indicated that emotion induction may increase expressive signal strength and improve downstream detection performance, suggesting the practical value of integrating affect elicitation into conversational AI systems.

### Comparison with Prior Work

Our findings are consistent with prior research demonstrating the dominant predictive role of textual features in depression detection. Studies based on clinical interview transcripts have shown that linguistic content often outperforms acoustic and visual modalities in predictive accuracy [8-10,34,35]. Our ablation results support this observation, as removing word-level embeddings resulted in the largest degradation in performance.

Compared with multimodal architectures, such as Yin et al [31], Zhang et al [14], and Li et al [16], our model achieves lower RMSE and MAE while maintaining a substantially smaller parameter footprint. While transformer-based or hierarchical recurrent frameworks emphasize deep cross-modal modeling, our attention-based fusion module demonstrates that efficient structured integration can achieve competitive or superior results with significantly reduced computational cost.

Relative to parameter-efficient text-only approaches such as Lau et al [32], our multimodal framework provides improved regression consistency, suggesting that incorporating behavioral cues beyond text enhances severity estimation. Additionally, compared with zero-shot LLMs (eg, GPT-4o, o3-mini), our model achieves stronger classification performance despite operating at a fraction of the parameter scale, highlighting the value of task-specific multimodal design over parameter scaling alone.

Finally, while psychological literature has established altered emotional reactivity in depression [11,12], few computational systems explicitly integrate emotion induction into multimodal detection pipelines. Our pilot findings suggest that embedding affect elicitation within conversational interaction may enhance behavioral signal salience in naturalistic settings.

### Limitation

This study has several limitations that warrant careful consideration. The real-world deployment involved a relatively small sample size (n=22), which constrains statistical power and limits the precision of effect size estimation. Although certain differences—particularly in the emotion-induction condition—reached statistical significance, the modest sample increases uncertainty regarding robustness and reproducibility. Larger-scale studies are necessary to confirm stability across broader populations.

Participants were primarily young adults affiliated with a university setting, resulting in a relatively homogeneous demographic profile. Such sampling may introduce selection bias and restrict external validity. Depression presentation varies across age groups, socioeconomic contexts, and cultural backgrounds. The generalizability of the current findings to older adults, clinical psychiatric populations, or cross-cultural environments, therefore, remains to be established.

PHQ-8 was used as the sole ground truth indicator of depressive symptom severity. While widely validated and frequently adopted in computational depression research, PHQ-8 is a self-report instrument rather than a clinician-administered diagnostic interview. Self-reported measures may be influenced by reporting bias, transient emotional states, or social desirability effects. Incorporating structured diagnostic interviews or multimethod assessment frameworks would strengthen clinical validity.

The emotion-induction protocol relied on a fixed set of emotionally evocative video stimuli. Emotional reactivity varies across individuals, and standardized stimuli may not elicit comparable affective responses in all participants. Although improved predictive performance was observed following induction, the study design does not fully disentangle whether the performance gain reflects increased emotional expressiveness, cognitive priming, or heightened engagement. More controlled experimental paradigms—including neutral baseline conditions or counterbalanced stimulus designs—would allow for clearer causal interpretation.

The data augmentation strategy also introduces considerations. Synthetic text samples were generated exclusively from training data to prevent information leakage; however, approximating PHQ-8 regression targets through threshold-based assignment reduces label granularity. This simplification may limit the precision of severity calibration. In addition, paraphrased transcripts, while semantically constrained, may differ distributionally from naturally occurring spontaneous conversations, potentially affecting generalization.

The evaluation framework focuses on cross-sectional assessment rather than longitudinal monitoring. Depression is dynamic and episodic, and a single interaction may not capture temporal symptom fluctuations. Long-term validation is needed to determine sensitivity to clinical change and suitability for continuous monitoring scenarios.

Finally, while attention mechanisms provide partial insight into modality weighting, the interpretability of specific multimodal feature contributions remains limited. Greater integration of explainability tools and clinician-in-the-loop evaluation would be necessary before large-scale clinical deployment.

### Future Directions

Future research should explore cross-cultural and multilingual generalization to broaden applicability across diverse populations. The emotion-induction mechanism may be further optimized through adaptive or reinforcement-based strategies to enhance user engagement.

In addition, incorporating continual and personalized learning mechanisms could support longitudinal adaptation without full retraining. Future work should also integrate explainability tools and conduct systematic failure case analyses to improve robustness and clinical interpretability. Finally, more controlled experimental designs, such as neutral-video conditions, are needed to better isolate emotion-specific induction effects.

### Conclusions

DEPRESAR-Fusion demonstrates that compact multimodal architectures can achieve state-of-the-art depression detection performance while remaining suitable for real-time SAR deployment. By integrating multimodal perception, lightweight deep fusion, and emotion-aware interaction mechanisms, this work advances practical, scalable, and field-validated approaches to AI-driven mental health support.

## Supplementary material

10.2196/84110 Multimedia Appendix 1 Mean user ratings on a 5-point Likert scale.

## Abbreviations

AI: artificial intelligence
AU: action unit
CCC: concordance correlation coefficient
DAIC-WOZ: Distress Analysis Interview Corpus-Wizard-of-Oz
DEPRESAR: Depression Social Assistant Robot
E-DAIC: Extended Distress Analysis Interview Corpus
eGeMAPS: extended Geneva Minimalistic Acoustic Parameter Set
ESC: emotional support conversation
LLM: large language model
MAE: mean absolute error
MFCC: Mel-frequency cepstral coefficient
PHQ-8: Patient Health Questionnaire-8
RMSE: root mean square error
SAR: socially assistive robot
