# Supplementary material for: Multimodal Depression Detection Through Conversational Interactions with an Emotion-Aware Social Robot: Pilot Study
Source: JMIR Form Res. 2026 Apr 27;10:e84110. doi: 10.2196/84110 (PMC13120753; doi:10.2196/84110)
Supplement: Multimedia Appendix 1 [file formative-v10-e84110-s001.docx]

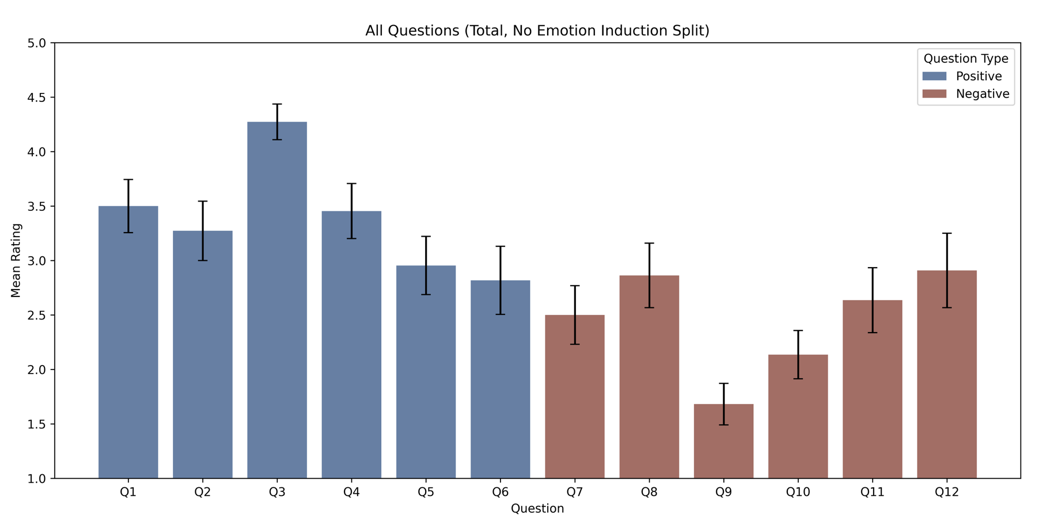


Figure 1: Mean user ratings (Q1-Q12) on a 5-point Likert scale. Error bars represent the SE of the mean. Q1-Q6 corresponds to positive statements (higher scores indicate better experience), whereas Q7-Q12 corresponds to negative statements (lower scores indicate better experience).
